# *Toxoplasma gondii* MAF1b Binds the Host Cell MIB Complex To Mediate Mitochondrial Association

**DOI:** 10.1128/mSphere.00183-17

**Published:** 2017-05-24

**Authors:** Felice D. Kelly, Brian M. Wei, Alicja M. Cygan, Michelle L. Parker, Martin J. Boulanger, John C. Boothroyd

**Affiliations:** a Department of Microbiology and Immunology, Stanford University School of Medicine, Stanford, California, USA; b Department of Biochemistry and Microbiology, University of Victoria, Victoria, British Columbia, Canada; University of Georgia

**Keywords:** *Toxoplasma gondii*, host-pathogen interactions, mitochondria

## Abstract

Many diverse intracellular pathogens, such as *Legionella pneumophila*, *Chlamydia psittaci*, *Encephalitozoon* sp., and *Toxoplasma gondii*, manipulate and relocate host cell organelles, including mitochondria. *Toxoplasma* tachyzoites use a secreted protein, mitochondrial association factor 1b (MAF1b), to drive the association between the host mitochondria and the membrane of the parasitophorous vacuole, in which the parasites grow. The identity of the host partner in this interaction, however, has not previously been identified. By exogenously expressing tagged MAF1b in mouse embryonic fibroblasts, we were able to isolate host cell proteins that specifically interact with MAF1b. We then verified these interactions in the MAF1b-expressing fibroblasts, as well as in the context of parasite infection in human fibroblasts and HeLa cells. The results show that a host cell mitochondrial complex, the mitochondrial intermembrane space bridging (MIB) complex, specifically interacts with MAF1b. We further demonstrate that a version of MAF1b that is deficient in host-mitochondrial association does not efficiently coprecipitate the MIB complex. Validation of the importance of the MAF1b-MIB interaction came from showing that knockdown of two MIB complex components, MIC60 and SAM50, substantially reduces mitochondrial association with the parasitophorous vacuole membrane. This interaction between a secreted membrane-integral parasite protein and a membrane-bound complex of a host organelle represents the first instance of organelle relocalization in which both the host and pathogen molecules are known and provides the foundation for more detailed biochemical studies.

**IMPORTANCE** Parasites interact intimately with their hosts, and the interactions shape both parties. The common human parasite *Toxoplasma gondii* replicates exclusively in a vacuole in a host cell and alters its host cell’s environment through secreted proteins. One of these secreted proteins, MAF1b, acts to concentrate mitochondria around the parasite’s vacuole, and this relocalization alters the host immune response. Many other intracellular pathogens also recruit host mitochondria, but the identities of the partners that mediate this interaction have not previously been described in any infection. Here, we show that *Toxoplasma* MAF1b binds to the multifunctional MIB protein complex on the host mitochondria. Reducing the levels of the proteins in this mitochondrial complex reduces the close association of host cell mitochondria and the parasite’s vacuole. This work provides new insight into a key host-pathogen interaction and identifies possible targets for future therapeutic intervention as well as a more molecular understanding of important biology.

## Abstract (precis)

Parasites interact intimately with their hosts, and the interactions shape both parties. The common human parasite *Toxoplasma gondii* replicates exclusively in a vacuole in a host cell and alters its host cell’s environment through secreted proteins. One of these secreted proteins, MAF1b, acts to concentrate mitochondria around the parasite’s vacuole, and this relocalization alters the host immune response. Many other intracellular pathogens also recruit host mitochondria, but the identities of the partners that mediate this interaction have not previously been described in any infection. Here, we show that *Toxoplasma* MAF1b binds to the multifunctional MIB protein complex on the host mitochondria. Reducing the levels of the proteins in this mitochondrial complex reduces the close association of host cell mitochondria and the parasite’s vacuole. This work provides new insight into a key host-pathogen interaction and identifies possible targets for future therapeutic intervention as well as a more molecular understanding of important biology.

## INTRODUCTION

Intracellular pathogens extensively manipulate their host cells to enable invasion (1), subvert the host immune response (2, 3), and make the host cell environment more conducive to pathogen growth. These manipulations include reprogramming of signaling and downstream gene expression (4–6), altering the cytoskeleton (7–9), modifying vesicle trafficking (10), and even rearranging host cell organelles, such as the lysosome, endoplasmic reticulum (ER), spindle-pole bodies, and mitochondria (11, 12). To effect these changes, bacteria and intracellular parasites secrete a diverse array of proteins into their host cells.

Manipulation of host mitochondria is common and may be a particularly advantageous adaptation as recent work has revealed that mitochondria are important not only for their traditional role in metabolism and energy production, but also as a location where innate immune signaling is concentrated (13). Despite the frequency of this phenomenon, the underlying mechanisms and associated consequences of it are incompletely understood. In the infections where the process has been studied most, the identity of either the host cell or pathogen binding partner is known, but not both. For example, *Chlamydia psittaci* inclusions are surrounded by host cell mitochondria (14) in a process that requires host cell kinesin activity (15). For the intracellular replicative vacuole of the microsporidian *Encephalitozoon cuniculi*, the mitochondrial porin VDAC is concentrated at the sites of contact between the vacuole and the host mitochondria (16). In the case of *Legionella pneumophila*, on the other hand, a purified bacterial chaperonin that localizes to the vacuole membrane is known to mediate the recruitment, but the host binding partner is not yet known (17).

As with these other pathogens, *Toxoplasma gondii* tachyzoites grow and divide only within a parasitophorous vacuole derived mostly from host cell lipids and formed during invasion. *Toxoplasma* successfully shapes this niche through the actions of an array of secreted effectors released from specialized organelles at different stages of invasion and growth. These effectors alter many aspects of host cell biology, including apoptotic signaling (18, 19), immune responses (20), and cellular architecture (21, 22). Early microscopic studies of *Toxoplasma* reported a close association between host cell mitochondria and the parasitophorous vacuolar membrane (PVM) (23, 24). Later observations revealed that this close association was not universal—of the three canonical clonal lineages of *Toxoplasma*, type I and type III strains display host cell mitochondrial association (HMA), whereas type II strains do not (25). Using the F1 progeny of a genetic cross between parasites that do and do not display HMA, a single multicopy locus was identified as necessary for mitochondrial association (25).

The *Toxoplasma* gene within this locus implicated in HMA encodes mitochondrial association factor 1b (MAF1b), a secreted effector protein that is expressed only in type I and III parasites and that localizes to contact sites between the PVM and host cell mitochondria (25). The multicopy locus that encodes MAF1b includes a family of up to 10 closely related paralogs in *Toxoplasma*, *Hammondia hammondi*, and *Neospora caninum*. Although these paralogs are highly similar in sequence, they differ in their ability to mediate HMA (25). For example, MAF1a paralogs are expressed in type II parasites but cannot mediate HMA (26). MAF1b has a signal peptide and a predicted transmembrane domain, but it has no homologous domains of known function. MAF1b is phosphorylated during host cell infection at multiple sites near the N and C termini of the protein, but the significance of this is not known (27). Deletion of the entire multicopy *MAF1* locus in type I parasites leads to a loss of HMA, and expression of a single copy of a type I allele of MAF1b in type II parasites induces HMA (25). Importantly, MAF1b that is exogenously expressed in mouse embryonic fibroblasts (MEFs) localizes to mitochondria, indicating that it is capable of directly binding host mitochondria even without other parasite proteins present (25). This provides an ideal system, therefore, for identification of the host binding partners of MAF1b. Here we show that MAF1b specifically associates with the host mitochondrial intermembrane space bridging (MIB) complex and show, using specific small interfering RNA (siRNA) knockdowns, that these interactions are important for *Toxoplasma*-mediated HMA.

## RESULTS

### *T. gondii* MAF1b exogenously expressed in mammalian cells coimmunoprecipitates components of the MIB complex.

MAF1b was previously shown to localize to the mitochondria of mouse embryonic fibroblasts (MEFs) when expressed as a transgene under the cytomegalovirus (CMV) promoter (25). In this system, unlike in a parasite infection, MAF1b is the only parasite protein present, which allows us to use these cells as a tool to identify potential host-interacting partners more easily. We used the N-terminal hemagglutinin (HA) tag on exogenously expressed MAF1b to coimmunoprecipitate MAF1b and bound host cell proteins, using nonengineered MEFs (MEFs not expressing MAF1b) as a negative control. Since MAF1b has a predicted transmembrane domain and many mitochondrial proteins are membrane bound, we performed the immunoprecipitation (IP) in buffers with low detergent levels, modified from other studies that characterized mitochondrial protein interactions (28).

**FIG 1:**
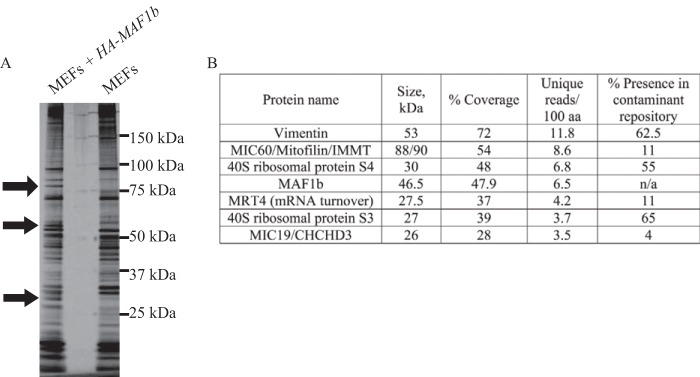
Identification of MAF1b-interacting partners. Protein lysate from confluent cultures of mouse embryonic fibroblasts (MEFs) exogenously expressing N-terminally HA-tagged MAF1b protein under a constitutive promoter (MEFs + *HA-MAF1b*) or MEF cells not expressing any exogenous proteins (MEFs) was incubated with anti-HA-conjugated beads. (A) Elutions from anti-HA immunoprecipitations resolved on a 4 to 12% bis-Tris gel and silver stained for total protein. Lane 1, MEFs exogenously expressing HA-MAF1b; lane 2, nonengineered control MEFs. Black arrows demarcate bands that were excised, pooled, and analyzed by LC-MS/MS. (B) Table of proteins identified from bands in panel A. Proteins with greater than 25% peptide coverage are listed, with obvious (nonmouse, non-MAF1b) contaminants excluded. The right column indicates each protein’s representation in an IP contaminant repository (see Materials and Methods).

After eluting bound proteins by adding HA-tagged peptide, we resolved the eluates on a polyacrylamide gel and stained with silver to identify specifically eluted proteins (Fig. 1A). As expected from a single-step purification, many proteins were present from both HA-MAF1b-expressing and nonengineered MEFs; three bands, however, were specifically present or enriched in the HA-MAF1b eluate, as indicated with arrows in Fig. 1A. We excised these bands and pooled them for analysis by liquid chromatography-tandem mass spectrometry (LC-MS/MS).

The results of the LC-MS/MS showed relatively few proteins that met the criterion of high peptide coverage (>25%). The complete list of identified proteins can be found in Table S1 in the supplemental material. The table in Fig. 1B lists the top hits from the submitted bands ranked by the ratio of unique peptides per 100 amino acids in the protein. We have listed all the proteins where total peptide coverage was greater than 25%. MAF1b was readily identified, as expected. Also included in this list were three proteins—vimentin and ribosomal proteins S3 and S4—that are commonly present in negative controls for such MS/MS analyses and are likely contaminants (29). In addition to these, there were three proteins—MRT4 (a nuclear protein involved in mRNA turnover), MIC60 (also known as mitofilin and IMMT), and MIC19 (Also known as CHCHD3)—that are not common, nonspecific molecules found in MS/MS analyses. MAF1b expressed in MEFs is found in various amounts in the nucleus (25), and the IP of MRT4 might be indicative of a true interaction, but this was not further pursued. Instead, given our goal of understanding the nature of the interaction of MAF1b with mitochondria, we focused on the two mitochondrial proteins, MIC60 and MIC19. These partners interact in a well-described complex called the mitochondrial contact site (MICOS) complex that localizes to the inner mitochondrial membrane (IMM) at sites where mitochondrial cristae contact the outer mitochondrial membrane (OMM) (30, 31). When this IP and MS/MS experiment was repeated, MRT4, MIC60, and MIC19 were all identified again in the second experiment (data not shown).

10.1128/mSphere.00183-17.1 TABLE S1  Full list of proteins identified by LC-MS/MS from excised bands in Fig. 1. Only nonmouse contaminant proteins were excluded from this list; proteins are ranked by log probability score. When multiple isoforms of a single protein are identified, the other metrics for the protein cannot be determined for each isoform, so those cells are left intentionally blank. Download TABLE S1, DOCX file, 0.1 MB. Copyright © 2017 Kelly et al. 2017 Kelly et al. This content is distributed under the terms of the Creative Commons Attribution 4.0 International license.

**FIG 2:**
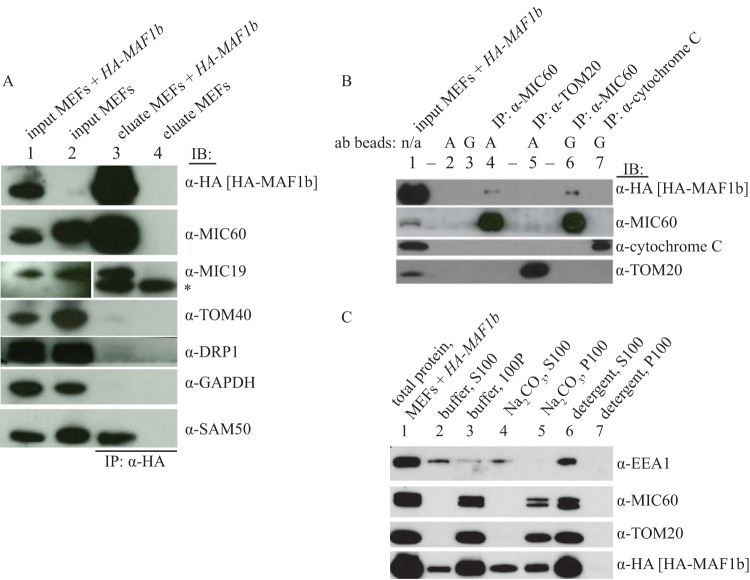
MAF1b interacts with the MIB complex in MEFs. (A) Immunoprecipitation of MEF lysates with anti-HA as described in Fig. 1 and analyzed by Western blotting. Inputs represent 1% of the starting material relative to the amount loaded from the eluates; immunoblotting (IB) was performed with the indicated antibodies. For anti-MIC19, the input lanes shown are from a shorter exposure than the elution lanes from the same gel and the asterisk indicates cross-reactivity with antibody light chain present as a contaminant in the eluates from the antibody column. This cross-reactivity was verified by probing the blot with secondary antibody alone (data not shown). For all other antibodies, the exposure for the input and the eluates was the same. Shown is one representative experiment from at least 5 biological replicates. (B) Reciprocal co-IP of mitochondrial proteins. Antibodies used for IP (listed above the blot) were bound to protein A or protein G beads (as indicated for each antibody and for negative controls). Antibody beads (ab beads) were then incubated with lysates from HA*-MAF1b* MEFs for ~18 h. The input (lane 1) represents 1% of the starting material used for the eluates. Lanes 2 and 3 show that none of these proteins is precipitated by protein A or protein G beads alone. A representative blot from one of two biological replicates is shown. (C) HA-MAF1 expressed in MEFs is partially extracted by carbonate. Shown are results from Western blot analysis of total cell lysates prepared from MEFs expressing HA-MAF1, treated as follows before fractionation and spinning at 100,000 × *g* for 1 h to produce a supernatant (S100) and pellet (P100), as indicated. Lane 1, no treatment, total protein; lanes 2 and 3, neutral buffer, no detergent; lanes 4 and 5, alkaline carbonate extraction (0.1 M Na_2_CO_3_ [pH 11.5]); lanes 6 and 7, detergent (1% Triton X-100, 0.1% SDS). Equal proportions of input were loaded in each lane. Shown are representative data from >3 independent experiments.

To test the validity of the coimmunoprecipitation (co-IP) of MIC60 and MIC19 with MAF1b, we repeated the IPs and evaluated the specificity via Western blotting, using nonengineered MEFs as a control for nonspecific binding. The results (Fig. 2A) showed that MAF1b selectively coimmunoprecipitated both MIC60 and MIC19 from the MAF1b-expressing MEFs. Notably, neither the abundant cytosolic protein GAPDH (glyceraldehyde-3-phosphate dehydrogenase) nor the OMM proteins TOM40 and DRP-1 coimmunoprecipitated, indicating that the coimmunoprecipitation of MIC60 and MIC19 with MAF1b is not the result of the immunoprecipitation of all mitochondrial proteins and that the co-IP process effectively removed an abundant cytosolic protein.

While the identification of MIC60 and MIC19 as binding partners of MAF1b is consistent with MAF1b binding host mitochondria, it was surprising since both MIC60 and MIC19 are IMM proteins. This suggested that either MAF1b spans the OMM and directly interacts with one or the other of these two MICOS complex members or that it binds a third protein in the OMM that acts as a bridge between MAF1b and MICOS. Indeed, MICOS is part of the larger mitochondrial intermembrane space bridging (MIB) complex (32, 33), which includes proteins that span the IMM and OMM. Thus, we hypothesized that an OMM component of the MIB complex might be the direct partner of MAF1b. A candidate for such a protein is SAM50, an OMM-spanning β-barrel protein with a large N-terminal domain in the intermembrane space (34). SAM50 binds other members of the MIB complex, including MIC60, and is essential for maintenance of cristae (33, 35, 36). To determine if SAM50 might be serving the bridging function, we analyzed the anti-HA immunoprecipitate for coprecipitation of SAM50. The results (Fig. 2A) show that SAM50 was indeed strongly enriched in the IP eluate from HA-MAF1b-expressing MEFs compared to MEFs that do not express HA-MAF1b. This strong co-IP of three members of the same complex led us to conclude that, at least when expressed in MEFs, MAF1b likely interacts with the MIB complex, and this is possibly through direct binding to SAM50, which is exposed on the OMM and therefore more easily accessible to MAF1b.

**FIG 3:**
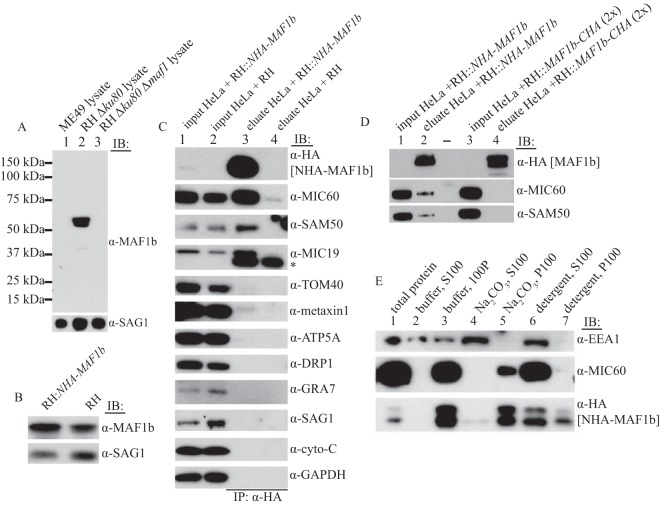
MAF1b interacts with the MIB complex in parasite-infected cells. (A) Rabbit anti-MAF1b antibody is specific for MAF1b. Lysates from type II ME49 parasites (which express MAF1a but not MAF1b [lane 1]), RH Δ*ku80* parasites (which express MAF1a and MAF1b [lane 2]), and RH Δ*ku8* Δ*maf1* parasites (missing the entire *MAF1* locus [lane 3]) were analyzed by Western blotting. Blots were probed with rabbit anti-MAF1b serum to assess reactivity and the parasite surface antigen anti-SAG1 to assess parasite protein loading. (B) Total MAF1 expression level in RH::NHA*-MAF1b* and RH parasites. Protein lysates from equal numbers of parasites were probed with antibodies against MAF1 to measure the total level of MAF1 protein and SAG1 as a parasite loading control. When the MAF1b protein levels are compared to the loading control, the additional copy of NHA-MAF1b results in a total MAF1b level of <2× wild type. (C) NHA-MAF1 from parasites coimmunoprecipitates with the MIB complex. Shown are the results from immunoprecipitation with anti-HA of lysate from HeLa cells infected with RH::NHA*-MAF1b* or the parental control RH strain for 18 h. Input and eluate were analyzed by Western blotting as in Fig. 2, except inputs represent 0.25% of the total starting material used for the eluates. A representative blot from at least 5 independent experiments is shown. (D) The interaction of MAF1b with the MIB complex is blocked by a C-terminal HA tag. Immunoprecipitation was performed with anti-HA of lysate from uninfected HeLa cells or HeLa cells infected with either RH::NHA*-MAF1b* or RH::*MAF1b*-CHA for 16 h. Input represents 0.2% of the total starting material used for the eluate for both infection conditions, but twice as much input and eluate material was added from the RH::*MAF1b*-CHA infection to normalize the fusion protein levels. A representative blot from two experiments is shown. (E) MAF1b is an integral membrane protein. Shown are results from Western blot analysis of total cell lysates prepared from HFF cells infected with *Toxoplasma* RH::NHA*-MAF1b* for ∼24 h and treated as follows before fractionation and spinning at 100,000 × *g* for 1 h to produce a supernatant (S100) and pellet (P100), as indicated. Treatment conditions were as described for Fig. 2. Lane 1, no treatment, total protein; lanes 2 and 3, neutral buffer, no detergent; lanes 4 and 5, alkaline carbonate extraction; lanes 6 and 7, detergent. Equal proportions of input were loaded in each lane. Shown are representative data from 3 independent experiments.

To further confirm the MAF1b-MIB interaction, we performed a reciprocal co-IP using antibodies raised against MIC60 and, as negative controls, antibodies raised to two mitochondrial proteins that are not part of the MIB complex, TOM20 and cytochrome *c* (Fig. 2B). The results showed that only the anti-MIC60 antibody coimmunoprecipitated HA-MAF1b (Fig. 2B, lanes 4 and 6). (Note that, although the co-IP of HA-MAF1b with anti-MIC60 was modest, it was reproducible and highly specific.) We were unable to perform similar experiments using commercial antibodies to MIC19 and SAM50 because, while these efficiently detected their target proteins in Western blots (e.g., Fig. 2 and 3), in our hands they did not robustly immunoprecipitate their respective targets (data not shown), precluding their use in IP-type experiments.

To interrogate more precisely how MAF1b is associating with the mitochondria, we performed a carbonate extraction of MEFs expressing HA-MAF1b. Carbonate extraction distinguishes between peripheral and integral membrane proteins since the former will unfold and lose their membrane association in the presence of 0.1 M Na_2_CO_3_ at high pH, while the latter will remain integrated into membranes. As controls, we used MIC60 and TOM20 as representative proteins that are known to be transmembrane proteins and EEA1 as a representative protein that is known to be a peripheral membrane protein. To control for protein insolubility, the Na_2_CO_3_ treatment was compared to protein extraction by detergent, which will disrupt the membrane and disassociate both peripheral and integral membrane proteins but not precipitated insoluble proteins. MIC60 and TOM20 were both insoluble in carbonate and were fully solubilized by detergent, as expected for membrane integral proteins (Fig. 2C). EEA1 was partially released from the membrane pellet after incubation in buffer, perhaps due to agitation, but was fully solubilized by sodium carbonate, as expected for a membrane peripheral protein. HA-MAF1b was partially solubilized after incubation with sodium carbonate, with approximately equal amounts of the protein found in the supernatant and membrane pellet (Fig. 2C).

The partial solubilization of MAF1b suggests that either MAF1b is associating with the mitochondria through very strong protein-protein interactions that are not fully disrupted by the sodium carbonate extraction or that there are two populations of protein present when it is exogenously expressed in MEFs: one that is membrane peripheral and one that is membrane integral. These two populations could come from differently localized HA-MAF1b, since the protein is observed to localize predominantly to the mitochondria but is also present in the cytoplasm and nucleus (25). The protein input for the carbonate extraction is depleted of nuclei and unbroken cells by an initial 800 × *g* spin, and then a membrane-enriched pellet is isolated by a 22,000 × *g* spin, so the levels of nuclear and cytoplasmic HA-MAF1b will be reduced but are probably still detectable. Alternatively, the two populations of MAF1b could both be present on the mitochondria and could be the result of stochastic spontaneous or directed insertion of the predicted transmembrane domain into nearby membranes (37, 38). We cannot currently distinguish among these possibilities.

Although these experiments were performed in an artificial expression system, this simplified system allowed us to identify a limited set of candidate proteins that we queried in immunoprecipitation experiments from parasite-infected host cells.

### MAF1b also interacts with the MIB complex during parasite infection.

To validate the results from the exogenous MEF expression system in the context of *Toxoplasma*-infected cells, we infected HeLa cells with type I RH tachyzoites expressing N-terminally tagged HA-MAF1b (NHA-MAF1b) expressed off the MAF1b promoter and repeated the IP studies. To test the expression level of this construct compared to the native protein, we generated rabbit antibody against MAF1b. It was previously reported that the type II ME49 strain does not recruit host cell mitochondria and does not express MAF1b, although it expresses the MAF1a paralog. As shown in Fig. 3A, the antibody that we generated against the C-terminal region of MAF1b, which is the most variable region, does not react with any protein from ME49, demonstrating its specificity for the MAF1b paralog. As expected the MAF1b band is absent from *ΔMAF1* lysates, made from parasites in which the entire *MAF1* gene cluster has been deleted (25). We used this antibody to evaluate the relative expression of MAF1b in wild-type RH and RH::NHA-*MAF1b* parasites. The expression level indicates that one extra copy is likely to be present, since the transgenic parasites express less than double the amount of total MAF1 of wild-type parasites (Fig. 3B).

Using these parasites, we infected HeLa cells for 18 h and then extracted protein and immunoprecipitated NHA-MAF1b with anti-HA antibodies. As a negative control, we also infected HeLa cells with RH parasites that express only the native, untagged MAF1b. As in the exogenously expressing MEFs, NHA-MAF1b from parasites strongly coimmunoprecipitated MIC60, SAM50, and MIC19 but did not coimmunoprecipitate the abundant cytosolic protein GAPDH, the parasite proteins GRA7 and SAG1, the outer mitochondrial membrane proteins TOM40, metaxin 1, and DRP1, or the inner mitochondrial proteins ATP5A or cytochrome *c* (Fig. 3C). The IP of SAM50 and MIC60, core proteins of the MIB complex, was robust and strong. The IP of NHA-MAF1b did not enrich for the OMM proteins TOM40, metaxin 1, and DRP1 nearly as strongly as it enriched for the core MIB components, again demonstrating specificity (Fig. 3C). These results are consistent with the association of MAF1b being specific for the MIB complex in *Toxoplasma*-infected cells. Ideally, we would have verified this interaction using an untagged MAF1b, but immunoprecipitation using the polyclonal antibody generated against MAF1b (Fig. 3A) did not yield coimmunoprecipitation of the MIB complex, as is often the case when polyclonal antibodies bind epitopes required for other intermolecular interactions (data not shown).

To further support the specificity of the interaction of MAF1b and the MIB complex and to ensure that it is not due to an artifact of the HA tag or any other aspect of the pulldown, we also tested coimmunoprecipitation of the MIB complex using parasites expressing C-terminally HA-tagged MAF1b (MAF1b-CHA). We previously reported that addition of an HA tag to the C terminus of MAF1b results in PVM localization of MAF1b, but the resulting protein is not capable of mediating HMA (25). The molecular basis of this disruption was unknown, but the data suggest that the C-terminal tag on MAF1b blocks mitochondrial association by interfering with interactions with the relevant host cell protein(s). We tested this by comparing NHA-MAF1b and MAF1b-CHA proteins for their ability to coimmunoprecipitate the MIB complex (Fig. 3D). The results showed that MIC60 and SAM50 more efficiently coimmunoprecipitate with NHA-MAF1b than with MAF1b-CHA. These data provide strong evidence that the interaction between MAF1b and the MIB complex is specific, biologically relevant, and dependent on accessibility of the C terminus of MAF1b, as previously argued (25).

### MAF1b is an integral membrane protein in parasite-infected host cells.

Although the MAF1b protein sequence is predicted to include a transmembrane (TM) domain and MAF1b is known to localize to the PVM-mitochondrial interface, it is unknown if MAF1b is, in fact, an integral membrane protein. To investigate this, we performed a carbonate extraction of membranes from parasite-infected human foreskin fibroblasts (HFFs) and probed these for the presence of MAF1b, as we had for HA-MAF1b exogenously expressed in MEFs (Fig. 2C). The results (Fig. 3E) showed that NHA-MAF1b was found almost entirely in the membrane pellet when infected cell lysates were treated with Na_2_CO_3_ (Fig. 3E, lane 5), indicating that it is generally associated with membranes in infected cells. MIC60 showed the same behavior, while the membrane-associated protein EEA1 was extracted by Na_2_CO_3_ (Fig. 3E, lane 4), as previously reported (39, 40). In the presence of detergent (Fig. 3E, lanes 6 and 7), MIC60 and EEA1 were fully extracted, whereas MAF1b was only partially extracted, consistent with an aggregated or insoluble subpopulation. Nevertheless, these results indicate that the bulk of MAF1b is likely an integral membrane protein in a parasite-infected cell, although they do not distinguish whether it is integrated into the PVM or the OMM.

### Assessment of binding using recombinant proteins.

Definitive confirmation of an interaction between two proteins can benefit from biochemical and/or structural experiments on recombinant proteins in isolation of all other proteins that might be serving as intermediaries. For this reason, we attempted to express full-length recombinant MAF1b (rMAF1b). Despite repeated attempts, however, we were able to express only the C-terminal ~2/3 of the protein (i.e., from amino acids 173 to 443 and omitting the predicted TM domain and extreme N-terminal portion) in the soluble fraction. We therefore attempted to demonstrate the ability of this rMAF1b to bind glutathione *S*-transferase (GST)-MIC60 under conditions similar to those used here for the IPs. No interaction between these two proteins was observed under any condition (data not shown). Unfortunately, we were unable to test the possibility of direct interaction between rMAF1b and recombinant SAM50 because SAM50 is a multipass integral membrane protein that is poorly expressed *in vitro* (34). Since we did not observe a specific interaction between rMAF1b and any of the MIB complex components from host cell lysate (data not shown), we did not pursue further experiments to test *in vitro* binding. Thus, we were unable to directly demonstrate which component of the MIB complex is the true binding partner of MAF1b.

### *Toxoplasma gondii* recruitment of host cell mitochondria is reduced after partial knockdown of MIB complex components.

**FIG 4:**
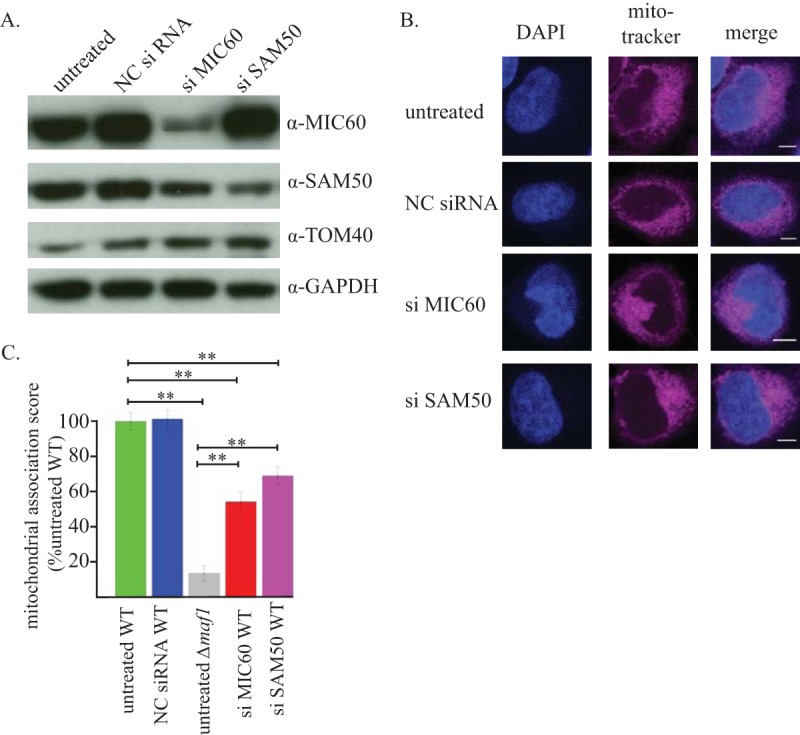
Knockdown of MIB complex components reduces mitochondrial association. (A) Western blot analysis of knockdown efficiency. Total cell lysates 72 h after HeLa transfection with siRNAs were analyzed by SDS-PAGE and Western blotting. The blot was probed with the indicated antibodies. NC, noncoding scramble siRNA control. (B) Mitochondrial morphology was not obviously altered by the short knockdown of MIC60 and SAM50. The images show uninfected fixed cells stained with DAPI and MitoTracker. Scale bars, 5 μm. (C) Analysis of the proportion of parasite vacuoles that show host mitochondrial association. Each vacuole was scored as follows: 2 for complete HMA (>80%), 1 for partial HMA (~80 to ~10%), or zero for no HMA (<10%). An investigator blind to sample identity scored 65 vacuoles per condition over two coverslips; scores were normalized, setting the average score for wild-type parasites infecting untreated cells as 100. **, *P* < 0.001 in pairwise comparisons. The difference in recruitment scores between the SAM50 and MIC60 siRNA treatments was not statistically significant. This experiment was repeated independently twice with similar results; one representative experiment is shown.

To validate the interaction between MAF1b and the MIB complex in a more biologically relevant context, we used siRNAs to knock down the levels of SAM50 and MIC60 and then assessed mitochondrial association with the PVM in *Toxoplasma-*infected HeLa cells. Forty-eight hours after transfection of the siRNA oligonucleotides in uninfected HeLa cells, we observed modest knockdown (53% reduction) of MIC60 and SAM50 (40% reduction) compared to protein levels in cells transfected with a noncomplementary RNA (ncRNA) (Fig. 4A). Note that, as previously published, the reduction in MIC60 protein levels by the addition of siRNA was accompanied by a reduction in SAM50 protein levels, whereas knockdown of SAM50 did not affect MIC60 protein levels (41). Long-term knockdown of SAM50 causes mitochondrial fragmentation (33), and long-term knockdown of MIC60 alters mitochondrial cristae (42), but gross mitochondrial morphology was not disrupted in the relatively short time frame of this experiment (72 h total) and with the partial knockdown of these proteins, as shown by the images of uninfected cells (Fig. 4B). Mitochondrial potential was also retained, as the potential-sensitive dye MitoTracker still efficiently stained the mitochondria 72 h after treatment.

Having successfully knocked down components of the MIB complex, we next assessed mitochondrial recruitment by microscopy. The siRNA-treated cells were infected for 24 h with *Toxoplasma* expressing cytoplasmic mCherry, and mitochondrial recruitment was assessed by microscopy using the cytoplasmic fluorescence of the parasites and Deep-Red MitoTracker (a version of MitoTracker that can be spectrally distinguished from mCherry and retains its fluorescence after fixation). Mitochondrial recruitment was assessed by capturing images of parasites and mitochondria in cells that contained parasitophorous vacuoles with at least two parasites inside. Because these parasites will, for the most part, have invaded the host at least 6 h prior to fixation (since parasites are more competent to invade in G_1_ [43], and one round of replication takes ~6 to 7 h for RH parasites [44]), we know that these parasites will have had sufficient time to establish HMA (25). Images were then scored for HMA by an investigator who was blind with regard to sample identity and could not distinguish between the samples based on mitochondrial morphology.

Each vacuole was scored as falling into one of three HMA categories: (i) vacuole largely surrounded (>80%) by mitochondria, (ii) vacuole partially surrounded by mitochondria (~80 to ~10%), and (iii) no apparent concentration of mitochondria around the vacuole (<10%). Average scores were then calculated and normalized to the mitochondrial association in untreated cells infected with wild-type parasites. As shown in Fig. 4C, when MIC60 or SAM50 was knocked down, the average mitochondrial recruitment score was significantly reduced compared to that of control cells (wild-type infected and untreated): i.e., 54% and 69% for the MIC60 and SAM50 siRNA-treated cells relative to untreated cells, respectively. While we cannot exclude the possibility that the inhibition of another mitochondrial function inhibits mitochondrial recruitment, these results are consistent with the biochemical data showing association of MAF1b and the MIB complex and provide evidence that this interaction is functionally relevant to the HMA phenomenon in infected cells.

## DISCUSSION

The manipulation of host cell organelles is a common strategy among pathogens, but the mechanisms of those manipulations are largely not understood. In this work, we show that the interaction between a secreted parasite protein, MAF1b, and a highly conserved host cell complex, the MIB complex, is important for the close apposition of the parasitophorous vacuole of *Toxoplasma gondii* and host cell mitochondria. While we were unable to definitively identify the direct binding target of MAF1b, the data here suggest the following model: MAF1b is a PVM protein that interacts with an OMM member of the MIB complex, possibly SAM50, and strong interactions within the complex result in the coimmunoprecipitation of MIC60 and MIC19.

We compared the interactions of the MIB complex with N- and C-terminally tagged MAF1b and showed that NHA-MAF1b coimmunoprecipitated MIC60 and SAM50 more efficiently than MAF1b-CHA. This suggests that the surface of MAF1b that interacts with the MIB complex is near the protein’s C terminus, although we cannot exclude the possibility that C-terminal tagging affects the overall structure of the MAF1b-CHA fusion protein. Both the N- and C-terminally tagged copies of MAF1b are present as second copies of the protein, so the native MAF1b is present in the cell as well; yet, the MAF1b-CHA acts as a dominant-negative protein during infection and blocks mitochondrial recruitment (25). This suggests that MAF1b acts as a dimer or multimer, a suggestion supported by the protein’s migration under nondenaturing conditions (data not shown), and that within the multimer some cooperativity is required to mediate HMA.

The functional importance of the MAF1b-MIB interaction is supported by the reduction in HMA that occurs when we reduce protein levels of MIC60 and SAM50 using siRNAs. For critical proteins like these, assessment of a role in a secondary function (e.g., HMA) is complicated by possible effects of their reduction on their primary function (i.e., SAM50’s role in protein import and MIC60’s role in mitochondrial morphology). In yeast, SAM50 is an essential gene, and it is thought to be likely essential in mammalian cells (45). Knockdown of SAM50 eventually causes mitochondrial fragmentation (33). Our efforts to generate a clustered regularly interspaced short palindromic repeat (CRISPR) knockout of MIC60 in HFFs were unsuccessful, which is not surprising since more complete knockdown of MIC60 increases apoptosis and decreases the growth rate of HeLa cells (42). Thus, the depletion of SAM50 and MIC60 needs to be carefully controlled, but partial depletion over a relatively short time scale allowed us to effectively assess the effect on HMA without drastically disrupting the mitochondria themselves.

Although the mechanism is unknown, recent work has shown that mitochondrial recruitment can confer a small growth advantage to *Toxoplasma in vivo* (26). While the work here does not directly address the question of why HMA confers a growth advantage, it is possible that binding of SAM50 and the MIB complex alters the interior structure of the mitochondria, resulting in a loss of connected IMM cristae (36, 42). The interaction between MAF1b and the MIB complex could alter the function of these complexes or overall mitochondrial function. Earlier work showed that mitochondria that are recruited to the parasitophorous vacuole have a greater cross-sectional area (25). Perhaps the interaction of MAF1b could stabilize the MIB complex in infected cells and induce mitochondrial fusion, which could subtly enhance parasite growth.

The interactions between pathogen proteins and host cell proteins shape the physiology of both organisms. Many interesting questions remain about how mitochondrial association with the parasitophorous vacuole membrane affects *Toxoplasma* and its host. The interactions described here will allow specific disruption of mitochondrial recruitment and lay the groundwork for a more detailed analysis of the mechanisms of mitochondrial dynamics. This work also sets the stage for structural studies of the molecular interaction between MAF1b and the MIB complex.

## MATERIALS AND METHODS

### Parasite strains, cell culture, and infections.

*Toxoplasma gondii* strains were maintained by growth in confluent primary human foreskin fibroblasts (HFFs) in Dulbecco’s modified Eagle’s medium (DMEM) (Invitrogen, Carlsbad, CA) with 10% fetal bovine serum (FBS; HyClone, Logan, UT), 2 mM glutamine, 100 U/ml penicillin, and 100 μg/ml streptomycin (cDMEM) at 37°C with 5% CO_2_. The following type I strains were used: RH Δ*hpt* (46), ME49, RH Δ*hpt* NHA*-MAF1b* (MAF1b tagged with a single HA epitope at the N terminus of the mature protein—i.e., just downstream of the predicted signal peptide [25]), RH Δ*hpt MAF1b-*CHA (MAF1b tagged with a single HA at its C terminus [25]), RH mCherry Δ*ku80* Δ*maf1* (25), and RH mCherry Δ*ku80* (47).

The MEF cell line expressing MAF1b with a single HA tag downstream of the signal peptide (HA-MAF1b) was previously described (25). H1 HeLa cells (CRL-1958 [ATCC]) were used for immunoprecipitations (IPs) from infected cells (Fig. 3).

### Immunoprecipitations.

Initial IPs to identify MAF1b-interacting proteins in MEFs were performed as follows. Four 15-cm dishes of MEFs for each condition (±HA-*MAF1b*) were grown to confluence. Cells were washed in cold phosphate-buffered saline (PBS) and then scraped in cell lysis buffer (50 mM Tris [pH 8.0], 150 mM NaCl, 1 mM phenylmethylsulfonyl fluoride [PMSF], 1% Igepal CA-630) supplemented with complete protease inhibitor cocktail (cOmplete, EDTA-free [Roche]). Cell lysate was repeatedly passed through a 27-gauge needle to break up cells and then subjected to sonication on ice (Branson Sonifier 250, with 3 sets of 10 s at 50% duty cycle and output control 2). Cell lysis was assessed by trypan blue staining. Cell lysates were spun at 800 × *g* for 5 min to remove insoluble material and unlysed cells. Lysates were then added to magnetic beads conjugated to anti-HA antibodies (Pierce) and incubated with gentle agitation for 4 h at 4°C. Unbound protein lysate was removed, and the anti-HA magnetic beads were then washed 5 times in cell lysis buffer. HA-MAF1b and HA-MAF1b bound proteins were eluted by competitive elution with 1 bed volume of HA peptide, and the eluate was resolved by electrophoresis on a 4 to 12% bis-Tris gel and stained with silver (48). Bands that were present only in the MEFs plus HA-*MAF1b* eluate were excised and pooled for mass spectrometric (MS) analysis.

### Mass spectrometric analysis.

For the MS, samples were digested with trypsin/lysC cocktail overnight (Promega) and the extracted peptides dried via SpeedVac. The liquid chromatograph (LC) was a Proxeon Easy nLC-II run at 300 nl/min. The column was in-house pulled and packed with 2.4-μm C_18_ material (Maisch GmbH High Performance LC), approximately 20 cm in length. Peptides were reconstituted in mobile phase A (0.525% acetic acid–water) and injected directly onto the analytical column of an LTQ-Orbitrap Velos set to acquire in data-dependent acquisition (DDA) mode, where the 15 most intense multiply charged precursor ions were selected for fragmentation in the ion trap, and the dynamic exclusion list was set to time out at 60 s. The .RAW data were searched by Byonic (49) against the canonical mouse database (with isoforms) from UniProt and filtered to a 1% false discovery rate (FDR) to generate a log probability score for each protein. The filtered results were further processed by EXCEL and MATLAB. The proteins with >25% peptide coverage were assessed for their presence in the contaminant repository for affinity purification-mass spectrometry data (29). This database does not have a contaminant repository for mouse proteins, so the human homolog of each MS-identified protein was used to estimate how likely a protein was to be a contaminant rather than a true binding partner.

### Immunoprecipitations and immunoblotting.

IPs to confirm the MS results were done as described above, but elution conditions were altered to give more consistent elution of HA-MAF1b. Elutions in Fig. 2 and 3 used a low-pH buffer to dissociate proteins from the antibody-conjugated beads. For Western blot analysis, protein samples were separated by SDS-PAGE and then transferred to polyvinylidene difluoride membranes. Membranes were blocked with 5% nonfat dry milk in PBS, and proteins were detected by incubation with primary antibodies diluted in PBS followed by incubation with secondary antibodies (raised in goat against the appropriate species) conjugated to horseradish peroxidase (HRP) and diluted in PBS unless otherwise stated. Antibody binding was detected with an enhanced chemiluminescence (ECL) kit (Pierce) and film. The following antibodies were used in Western blots: rat monoclonal anti-HA antibody directly conjugated to HRP (Roche, catalog no. ab 3F10 [diluted 1:1,000]), rabbit polyclonal anti-MIC60 (Proteintech, catalog no. 10179-1-AP [diluted 1:1,000]), rabbit monoclonal anti-SAM50 (Abcam, Inc., catalog no. AB167430 [diluted 1:5,000]), mouse polyclonal anti-MIC19 (Abcam, Inc., catalog no. AB69328 [diluted 1:1,000]), rabbit monoclonal anti-EEA1 (Cell Signaling, catalog no. 3288 [diluted 1:1,000]), rabbit polyclonal anti-TOM20 (Santa Cruz Biotechnology, catalog no. sc-11415 [diluted 1:1,000]), rabbit polyclonal anti-TOM40 (Santa Cruz Biotechnology, catalog no. sc-11414 [diluted 1:500]), mouse monoclonal anti-cytochrome *c* (Santa Cruz Biotechnology, catalog no. sc-13156 [diluted 1:1,000]), mouse monoclonal anti-GAPDH (Calbiochem, catalog no. CB-1001 [diluted 1:10,000]), mouse monoclonal anti-DRP-1 (Abcam, Inc., catalog no. AB56788 [diluted 1:1,000]), mouse anti-metaxin1 (Santa Cruz Biotech, catalog no. sc-514469 [diluted 1:1,000]), anti-ATP5A (Abcam, Inc., catalog no. AB14748 [diluted 1:1,000]), rabbit polyclonal anti-SAG1 (generated previously to recombinant SAG1 [diluted 1:20,000]), rabbit anti-GRA7 (generated previously [50] [diluted 1:10,000]), and rabbit polyclonal anti-MAF1b (generated in this study as described below [diluted 1:10,000 in PBS plus 5% milk]).

The conditions for the IPs from infected cells were described above. Host cells (HeLa) were infected for 16 to 24 h before protein harvest. Elutions were performed using a low-pH buffer. All relative protein quantification (Fig. 3B and 4A) from Western blots was performed using the “analyze gels” tool within ImageJ (version 1.45s).

### Generation of anti-MAF1b antibodies.

To generate a polyclonal anti-MAF1b antibody, we worked with Covance, Inc., using their 118-day antibody generation protocol. Briefly, after prescreening serum to exclude animals already reactive to *Toxoplasma*, a rabbit was injected with 250 μg of His_6_-tagged recombinant MAF1b (rMAF1b), prepared as described by Adomako-Ankomah et al. (26) and representing amino acids 173 through 443, emulsified in Freund’s complete adjuvant. The rabbit was boosted 5 times at 21-day intervals with 125 μg rMAF1b emulsified in Freund’s incomplete adjuvant. Antibody production was monitored with two test bleeds before sacrifice of the animal for a final production bleed at 118 days. Antibody specificity for MAF1b was evaluated by comparing the reactivities of the rabbit serum to *Toxoplasma* lysates from wild-type parasites and parasites with the *MAF1* locus deleted (Fig. 3A). Specificity for MAF1b was evaluated by comparing the reactivity of the rabbit serum to *Toxoplasma* lysates from type I RH-derived parasites and type II ME49-derived parasites (Fig. 3A). The animal usage was approved by the Institutional Animal Care and Use Committee at Stanford University. Covance, Inc., is accredited by the Association for Assessment and Accreditation of Laboratory Animal Care.

### Reciprocal co-IP.

Immunoprecipitating antibodies were incubated overnight with magnetic protein A or protein G beads (Pierce) and then washed in lysis buffer. Protein extracts from MEFs were then added to the antibody beads and incubated for 2 h at room temperature, and then the beads were washed 5 times in cell lysis buffer before bound proteins were eluted by boiling in 2× Laemmli buffer supplemented with dithiothreitol (DTT).

### Carbonate extraction.

Protein extracts from infected cells or from MEFs exogenously expressing HA-MAF1 were made by mechanical lysis in hypotonic buffer (10 mM NaCl, 1.5 mM MgCl_2_, 10 mM Tris HCl [pH 7.5]) without detergent. This lysate was split for three treatments. In the first treatment, lysates were incubated in the lysis buffer. In the second treatment, protein was incubated in lysis buffer adjusted to 0.1 M Na_2_CO_3_ (pH 11.5). In the third treatment, detergent (1% Triton X-100, 0.1% SDS) was added to the lysis buffer. Under all three conditions, proteins were incubated for 45 min on ice and then soluble proteins were separated from membrane-bound or insoluble proteins through a 1-h spin at 100,000 × *g*. This protocol was modified from that of Fujiki et al. (51).

### siRNA knockdown.

HeLa cells were plated on poly-l-lysine-coated coverslips in 24-well culture plates and transfected at ~50% confluence. Transfections of siRNA constructs were performed with DharmaFECT reagent (GE Dharmacon), per the manufacturer’s protocol. HeLa cells were incubated in siRNA mixture at 37°C for 24 h, after which medium was replaced with cDMEM and incubated at 37°C for an additional 24 h. The sequences of the targets of the siRNA constructs are as follows, as described previously (42, 52): MIC60, 5′-AAUUGCUGGAGCUGGCCUUTT-3′; Sam50, 5′-GGACATTCACTGAAATCATCT-3′.

### Evaluation of mitochondrial association.

Transfected and control cells were infected with RH Δ*hpt* or RH Δ*hpt* Δ*maf1* mCherry-expressing parasites at a multiplicity of infection of 1. Infections were allowed to proceed for 24 h, after which cells were either harvested for protein extraction or prepared for imaging. Cell lysates were prepared with radioimmunoprecipitation assay (RIPA) buffer (50 mM Tris, 150 mM NaCl, 0.1% SDS, 0.5% sodium deoxycholate, 1% Triton X-100, and complete protease inhibitor cocktail [cOmplete, EDTA-free; Roche]), and analyzed by Western blotting. MitoTracker Deep Red FM (Molecular Probes) was used to stain mitochondria according to the manufacturer’s protocol.

For imaging, cells were fixed in prewarmed serum-free cDMEM with 4% paraformaldehyde and 0.1% glutaraldehyde for 20 min at 37°C. The cells were then rinsed in PBS and mounted using Vectashield mounting medium with DAPI (4′,6-diamidino-2-phenylindole). A Zeiss LSM510 inverted confocal microscope was used to scan the cells. Images were acquired at room temperature with ZEN imaging software with a 63× objective lens.

Cell images were analyzed in the Fiji distribution of ImageJ. Vacuoles containing two or more parasites were assessed for mitochondrial recruitment. Vacuoles were visually scored as either 0 (<10% of the vacuole surrounded by mitochondria), 1 (10 to 80% of the vacuole surrounded by mitochondria), or 2 (>80% of the vacuole surrounded by mitochondria), depending on the amount of MitoTracker signal associated with the PVM relative to the rest of the cell. Sixty-five vacuoles per siRNA condition were scored by an investigator blind to sample identity. Mitochondrial association scores were normalized to the mean association score of the wild-type-infected untreated host cells, which was set at 100%. Pairwise *t* tests of mitochondrial recruitment scores were performed between each siRNA transfection condition to determine statistical significance. *P* values were adjusted by the Bonferroni method.
