# Supplementary material for: Toxoplasma gondii MAF1b Binds the Host Cell MIB Complex To Mediate Mitochondrial Association
Source: mSphere. 2017 May 24;2(3):e00183-17. doi: 10.1128/mSphere.00183-17 (PMC5444011; doi:10.1128/mSphere.00183-17)
Supplement: TABLE S1 [file sph003172288st1.docx]

| Table S1 | | | | | | | |
| --- | --- | --- | --- | --- | --- | --- | --- |
| Protein Description | \|Log Prob\| | # of spectra | # of unique peptides | unique peptides/AA's in protein | unique peptides/100 aa | Coverage % | # AA's in protein |
| \|Q8CAQ8\|IMMT_MOUSE Mitochondrial inner membrane protein | 74.91 | 88 | 65 | 0.0859 | 8.59 | 54.2 | 757 |
| \|Q8CAQ8-2\|IMMT_MOUSE Isoform 2 of Mitochondrial inner membrane protein |  |  |  |  |  |  |  |
| \|Q8CAQ8-5\|IMMT_MOUSE Isoform 5 of Mitochondrial inner membrane protein |  |  |  |  |  |  |  |
| \|P20152\|VIME_MOUSE Vimentin | 70.60 | 75 | 55 | 0.1180 | 11.80 | 72.1 | 466 |
| bait protein, HA-MAF1 | 40.28 | 59 | 28 | 0.0645 | 6.45 | 47.9 | 434 |
| \|Q9D0E1\|HNRPM_MOUSE Heterogeneous nuclear ribonucleoprotein M | 23.99 | 25 | 21 | 0.0288 | 2.88 | 24.6 | 729 |
| \|Q9D0E1-2\|HNRPM_MOUSE Isoform 2 of Heterogeneous nuclear ribonucleoprotein M |  |  |  |  |  |  |  |
| \|P62908\|RS3_MOUSE 40S ribosomal protein S3 | 19.51 | 11 | 9 | 0.0370 | 3.70 | 38.7 | 243 |
| \|P62702\|RS4X_MOUSE 40S ribosomal protein S4, X | 18.22 | 19 | 18 | 0.0684 | 6.84 | 47.9 | 263 |
| \|Q08943\|SSRP1_MOUSE FACT complex subunit SSRP1 | 16.15 | 14 | 11 | 0.0155 | 1.55 | 15.5 | 708 |
| \|Q08943-2\|SSRP1_MOUSE Isoform 2 of FACT complex subunit SSRP1 |  |  |  |  |  |  |  |
| \|Q03265\|ATPA_MOUSE ATP synthase subunit alpha, mitochondrial | 15.42 | 13 | 10 | 0.0181 | 1.81 | 16.8 | 553 |
| \|Q9QXS1\|PLEC_MOUSE Plectin | 14.69 | 14 | 12 | 0.0026 | 0.26 | 2.2 | 4691 |
| \|Q9QXS1-2\|PLEC_MOUSE Isoform PLEC-1 of Plectin |  |  |  |  |  |  |  |
| \|Q9QXS1-3\|PLEC_MOUSE Isoform PLEC-1A of Plectin |  |  |  |  |  |  |  |
| \|Q9QXS1-4\|PLEC_MOUSE Isoform PLEC-1B,2A of Plectin |  |  |  |  |  |  |  |
| \|Q9QXS1-5\|PLEC_MOUSE Isoform PLEC-1B of Plectin |  |  |  |  |  |  |  |
| \|Q9QXS1-6\|PLEC_MOUSE Isoform PLEC-0,1C of Plectin |  |  |  |  |  |  |  |
| \|Q9QXS1-7\|PLEC_MOUSE Isoform PLEC-0,1C,2A of Plectin |  |  |  |  |  |  |  |
| \|Q9QXS1-8\|PLEC_MOUSE Isoform PLEC-0,1C,2A,3A of Plectin |  |  |  |  |  |  |  |
| \|Q9QXS1-9\|PLEC_MOUSE Isoform PLEC-1D,2A of Plectin |  |  |  |  |  |  |  |
| \|Q9QXS1-10\|PLEC_MOUSE Isoform PLEC-1D of Plectin |  |  |  |  |  |  |  |
| \|Q9QXS1-11\|PLEC_MOUSE Isoform PLEC-1E,2A of Plectin |  |  |  |  |  |  |  |
| \|Q9QXS1-12\|PLEC_MOUSE Isoform PLEC-1E of Plectin |  |  |  |  |  |  |  |
| \|Q9QXS1-13\|PLEC_MOUSE Isoform PLEC-1F of Plectin |  |  |  |  |  |  |  |
| \|Q9QXS1-14\|PLEC_MOUSE Isoform PLEC-1G of Plectin |  |  |  |  |  |  |  |
| \|Q9QXS1-15\|PLEC_MOUSE Isoform PLEC-1H of Plectin |  |  |  |  |  |  |  |
| \|Q9QXS1-16\|PLEC_MOUSE Isoform PLEC-1I of Plectin |  |  |  |  |  |  |  |
| \|Q61753\|SERA_MOUSE D-3-phosphoglycerate dehydrogenase | 14.20 | 6 | 5 | 0.0094 | 0.94 | 11.1 | 533 |
| \|Q9D0I8\|MRT4_MOUSE mRNA turnover protein 4 homolog | 12.69 | 11 | 10 | 0.0418 | 4.18 | 37.2 | 239 |
| \|Q9QWL7\|K1C17_MOUSE Keratin, type I cytoskeletal 17 | 12.28 | 14 | 11 | 0.0254 | 2.54 | 19.4 | 433 |
| \|Q9CRB9\|CHCH3_MOUSE Coiled-coil-helix-coiled-coil-helix domain-containing protein 3, mitochondrial | 12.00 | 9 | 8 | 0.0352 | 3.52 | 27.8 | 227 |
| \|P62918\|RL8_MOUSE 60S ribosomal protein L8 | 11.14 | 9 | 9 | 0.0350 | 3.50 | 24.5 | 257 |
| \|P09405\|NUCL_MOUSE Nucleolin | 10.86 | 10 | 9 | 0.0127 | 1.27 | 11.3 | 707 |
| \|P47911\|RL6_MOUSE 60S ribosomal protein L6 | 9.86 | 7 | 6 | 0.0203 | 2.03 | 18.2 | 296 |
| \|P29341\|PABP1_MOUSE Polyadenylate-binding protein 1 | 7.05 | 10 | 8 | 0.0126 | 1.26 | 12.3 | 636 |
| \|P63101\|1433Z_MOUSE 14-3-3 protein zeta/delta | 6.56 | 6 | 4 | 0.0163 | 1.63 | 13.9 | 245 |
| \|P57784\|RU2A_MOUSE U2 small nuclear ribonucleoprotein A' | 5.91 | 6 | 6 | 0.0235 | 2.35 | 22.4 | 255 |
| \|P62806\|H4_MOUSE Histone H4 | 5.49 | 2 | 2 | 0.0194 | 1.94 | 17.5 | 103 |
| \|Q9ESX5\|DKC1_MOUSE H/ACA ribonucleoprotein complex subunit 4 | 5.07 | 5 | 2 | 0.0039 | 0.39 | 3.3 | 509 |
| \|P70696\|H2B1A_MOUSE Histone H2B type 1-A | 4.82 | 3 | 3 | 0.0236 | 2.36 | 18.1 | 127 |
| \|Q64475\|H2B1B_MOUSE Histone H2B type 1-B |  |  |  |  |  |  |  |
| \|P10853\|H2B1F_MOUSE Histone H2B type 1-F/J/L |  |  |  |  |  |  |  |
| \|Q8CGP1\|H2B1K_MOUSE Histone H2B type 1-K |  |  |  |  |  |  |  |
| \|Q8CGP2\|H2B1P_MOUSE Histone H2B type 1-P |  |  |  |  |  |  |  |
| \|Q8CGP2-2\|H2B1P_MOUSE Isoform 2 of Histone H2B type 1-P |  |  |  |  |  |  |  |
| \|Q99KP6\|PRP19_MOUSE Pre-mRNA-processing factor 19 | 4.72 | 4 | 3 | 0.0060 | 0.60 | 5.2 | 504 |
| \|Q99KP6-2\|PRP19_MOUSE Isoform 2 of Pre-mRNA-processing factor 19 |  |  |  |  |  |  |  |
| \|Q99KP6-3\|PRP19_MOUSE Isoform 3 of Pre-mRNA-processing factor 19 |  |  |  |  |  |  |  |
| \|P28656\|NP1L1_MOUSE Nucleosome assembly protein 1-like 1 | 4.68 | 4 | 4 | 0.0102 | 1.02 | 10.0 | 391 |
| \|Q8VEM8\|MPCP_MOUSE Phosphate carrier protein, mitochondrial | 4.30 | 3 | 3 | 0.0084 | 0.84 | 8.1 | 357 |
| \|Q922U2\|K2C5_MOUSE Keratin, type II cytoskeletal 5 | 4.15 | 6 | 6 | 0.0103 | 1.03 | 7.6 | 580 |
| \|P17225\|PTBP1_MOUSE Polypyrimidine tract-binding protein 1 | 3.86 | 5 | 4 | 0.0076 | 0.76 | 6.8 | 527 |
| \|P10126\|EF1A1_MOUSE Elongation factor 1-alpha 1 | 3.85 | 1 | 1 | 0.0022 | 0.22 | 2.4 | 462 |
| \|P62631\|EF1A2_MOUSE Elongation factor 1-alpha 2 |  |  |  |  |  |  |  |
| \|P07724\|ALBU_MOUSE Serum albumin | 3.68 | 1 | 1 | 0.0016 | 0.16 | 2.1 | 608 |
| \|Q3UV17\|K22O_MOUSE Keratin, type II cytoskeletal 2 oral | 3.61 | 3 | 2 | 0.0034 | 0.34 | 1.5 | 594 |
| \|O70251\|EF1B_MOUSE Elongation factor 1-beta | 3.45 | 2 | 2 | 0.0089 | 0.89 | 8.9 | 225 |
| \|Q91VN4\|CHCH6_MOUSE Coiled-coil-helix-coiled-coil-helix domain-containing protein 6 | 3.27 | 2 | 1 | 0.0037 | 0.37 | 2.9 | 273 |
| \|P63154\|CRNL1_MOUSE Crooked neck-like protein 1 | 3.03 | 3 | 2 | 0.0029 | 0.29 | 2.8 | 690 |
| \|P50446\|K2C6A_MOUSE Keratin, type II cytoskeletal 6A | 2.89 | 3 | 3 | 0.0054 | 0.54 | 5.4 | 553 |
| \|P01869\|IGH1M_MOUSE Ig gamma-1 chain C region, membrane-bound form | 2.85 | 3 | 2 | 0.0051 | 0.51 | 5.6 | 393 |
| \|P01868\|IGHG1_MOUSE Ig gamma-1 chain C region secreted form |  |  |  |  |  |  |  |
| \|Q62504\|MINT_MOUSE Msx2-interacting protein | 2.63 | 5 | 5 | 0.0014 | 0.14 | 0.7 | 3644 |
| \|Q62504-2\|MINT_MOUSE Isoform 2 of Msx2-interacting protein |  |  |  |  |  |  |  |
| \|Q62504-3\|MINT_MOUSE Isoform 3 of Msx2-interacting protein |  |  |  |  |  |  |  |
| \|P61982\|1433G_MOUSE 14-3-3 protein gamma | 2.58 | 4 | 3 | 0.0121 | 1.21 | 10.9 | 247 |
| \|Q7TMK9\|HNRPQ_MOUSE Heterogeneous nuclear ribonucleoprotein Q | 2.46 | 2 | 2 | 0.0032 | 0.32 | 3.2 | 623 |
| \|Q7TMK9-2\|HNRPQ_MOUSE Isoform 2 of Heterogeneous nuclear ribonucleoprotein Q |  |  |  |  |  |  |  |
| \|P10852\|4F2_MOUSE 4F2 cell-surface antigen heavy chain | 2.38 | 1 | 1 | 0.0019 | 0.19 | 1.5 | 526 |
| \|O35387\|HAX1_MOUSE HCLS1-associated protein X-1 | 2.31 | 1 | 1 | 0.0036 | 0.36 | 2.9 | 280 |
| \|Q62407\|SPEG_MOUSE Striated muscle-specific serine/threonine-protein kinase | 2.26 | 1 | 1 | 0.0003 | 0.03 | 0.2 | 3262 |
| \|Q62407-3\|SPEG_MOUSE Isoform 3 of Striated muscle-specific serine/threonine-protein kinase |  |  |  |  |  |  |  |
| \|Q62407-4\|SPEG_MOUSE Isoform 4 of Striated muscle-specific serine/threonine-protein kinase |  |  |  |  |  |  |  |
| \|P17156\|HSP72_MOUSE Heat shock-related 70 kDa protein 2 | 2.15 | 3 | 3 | 0.0047 | 0.47 | 5.7 | 633 |
| \|P63017\|HSP7C_MOUSE Heat shock cognate 71 kDa protein |  |  |  |  |  |  |  |
| \|Q9QYB1\|CLIC4_MOUSE Chloride intracellular channel protein 4 | 2.09 | 3 | 1 | 0.0040 | 0.40 | 2.4 | 253 |
| \|O88569\|ROA2_MOUSE Heterogeneous nuclear ribonucleoproteins A2/B1 | 2.08 | 1 | 1 | 0.0028 | 0.28 | 2.8 | 353 |
| \|Q8BJS4\|SUN2_MOUSE SUN domain-containing protein 2 | 1.98 | 1 | 1 | 0.0014 | 0.14 | 1.6 | 731 |
| >Reverse \|Q9JKS4\|LDB3_MOUSE LIM domain-binding protein 3 | 1.96 | 2 | 2 | 0.0028 | 0.28 | 2.9 | 723 |
| >Reverse \|Q8C7B8\|ZSWM4_MOUSE Zinc finger SWIM domain-containing protein 4 | 1.96 | 1 | 1 | 0.0009 | 0.09 | 0.5 | 1101 |
| \|Q61171\|PRDX2_MOUSE Peroxiredoxin-2 | 1.81 | 2 | 1 | 0.0051 | 0.51 | 5.6 | 198 |
| \|Q60930\|VDAC2_MOUSE Voltage-dependent anion-selective channel protein 2 | 1.81 | 2 | 1 | 0.0034 | 0.34 | 4.1 | 295 |
| \|Q62388\|ATM_MOUSE Serine-protein kinase ATM | 1.77 | 1 | 1 | 0.0003 | 0.03 | 0.4 | 3066 |
| \|P47963\|RL13_MOUSE 60S ribosomal protein L13 | 1.71 | 4 | 4 | 0.0190 | 1.90 | 15.6 | 211 |
| \|Q8BG05\|ROA3_MOUSE Heterogeneous nuclear ribonucleoprotein A3 | 1.67 | 1 | 1 | 0.0026 | 0.26 | 2.6 | 379 |
| \|Q91YU8\|SSF1_MOUSE Suppressor of SWI4 1 homolog | 1.62 | 4 | 4 | 0.0085 | 0.85 | 8.3 | 470 |
| \|Q61881\|MCM7_MOUSE DNA replication licensing factor MCM7 | 1.55 | 1 | 1 | 0.0014 | 0.14 | 1.8 | 719 |
| \|P60710\|ACTB_MOUSE Actin, cytoplasmic 1 | 1.51 | 2 | 2 | 0.0053 | 0.53 | 6.9 | 375 |
| \|Q3TEA8\|HP1B3_MOUSE Heterochromatin protein 1-binding protein 3 | 1.50 | 1 | 1 | 0.0018 | 0.18 | 1.6 | 554 |
| \|Q6IFX2\|K1C42_MOUSE Keratin, type I cytoskeletal 42 | 1.50 | 3 | 2 | 0.0044 | 0.44 | 3.1 | 452 |
| >Reverse \|Q80Z37\|TOPRS_MOUSE E3 ubiquitin-protein ligase Topors | 1.50 | 3 | 3 | 0.0029 | 0.29 | 1.9 | 1033 |
| \|Q8CGP5\|H2A1F_MOUSE Histone H2A type 1-F | 1.44 | 3 | 3 | 0.0231 | 2.31 | 20.8 | 130 |
| \|O70456\|1433S_MOUSE 14-3-3 protein sigma | 1.40 | 1 | 1 | 0.0040 | 0.40 | 3.2 | 248 |
| \|Q8BZ60\|STON2_MOUSE Stonin-2 | 1.40 | 1 | 1 | 0.0011 | 0.11 | 0.8 | 895 |
| >Reverse \|O08710\|THYG_MOUSE Thyroglobulin | 1.37 | 1 | 1 | 0.0004 | 0.04 | 0.4 | 2766 |
| \|Q9JMB8\|CNTN6_MOUSE Contactin-6 | 1.33 | 1 | 1 | 0.0010 | 0.10 | 1.3 | 1028 |
| \|Q61781\|K1C14_MOUSE Keratin, type I cytoskeletal 14 | 1.30 | 2 | 1 | 0.0021 | 0.21 | 1.5 | 484 |
| \|Q6DFV3\|RHG21_MOUSE Rho GTPase-activating protein 21 | 1.28 | 3 | 2 | 0.0010 | 0.10 | 0.8 | 1944 |
| >Reverse \|P98083\|SHC1_MOUSE SHC-transforming protein 1 | 1.22 | 22 | 1 | 0.0017 | 0.17 | 1.4 | 579 |
| \|Q6JHY2\|SMGC_MOUSE Submandibular gland protein C | 1.21 | 1 | 1 | 0.0014 | 0.14 | 1.5 | 733 |
| >Reverse \|Q923T7\|TRIM7_MOUSE Tripartite motif-containing protein 7 | 1.21 | 2 | 1 | 0.0020 | 0.20 | 1.2 | 510 |
| \|Q8K409\|DPOLB_MOUSE DNA polymerase beta | 1.19 | 1 | 1 | 0.0030 | 0.30 | 1.8 | 335 |
| >Reverse \|Q3UHN9\|NDST1_MOUSE Bifunctional heparan sulfate N-deacetylase/N-sulfotransferase 1 | 1.19 | 1 | 1 | 0.0011 | 0.11 | 1.5 | 882 |
| \|P55772\|ENTP1_MOUSE Ectonucleoside triphosphate diphosphohydrolase 1 | 1.19 | 1 | 1 | 0.0020 | 0.20 | 1.2 | 510 |
| \|Q8C753\|K0556_MOUSE Uncharacterized protein KIAA0556 | 1.17 | 1 | 1 | 0.0006 | 0.06 | 0.4 | 1610 |
| \|Q9JKB3\|DBPA_MOUSE DNA-binding protein A | 1.11 | 5 | 3 | 0.0083 | 0.83 | 6.9 | 361 |
| >Reverse \|Q8BP97\|RHBD3_MOUSE Rhomboid domain-containing protein 3 | 1.09 | 1 | 1 | 0.0026 | 0.26 | 1.8 | 385 |
| >Reverse \|P46467\|VPS4B_MOUSE Vacuolar protein sorting-associated protein 4B | 1.09 | 2 | 2 | 0.0045 | 0.45 | 3.4 | 444 |
| >Reverse \|Q8CF89\|TAB1_MOUSE TGF-beta-activated kinase 1 and MAP3K7-binding protein 1 | 1.07 | 1 | 1 | 0.0020 | 0.20 | 2.0 | 502 |
| \|A2AVA0\|SVEP1_MOUSE Sushi, von Willebrand factor type A, EGF and pentraxin domain-containing protein 1 | 0.88 | 2 | 2 | 0.0006 | 0.06 | 0.6 | 3567 |
| \|P70704\|AT8A1_MOUSE Probable phospholipid-transporting ATPase IA | 0.87 | 1 | 1 | 0.0009 | 0.09 | 0.4 | 1149 |
| >Reverse \|Q99JR8\|SMRD2_MOUSE SWI/SNF-related matrix-associated actin-dependent regulator of chromatin subfamily D member 2 | 0.85 | 1 | 1 | 0.0019 | 0.19 | 7.3 | 531 |
| \|Q3U1N2\|SRBP2_MOUSE Sterol regulatory element-binding protein 2 | 0.82 | 1 | 1 | 0.0009 | 0.09 | 0.6 | 1130 |
| \|P25444\|RS2_MOUSE 40S ribosomal protein S2 | 0.82 | 1 | 1 | 0.0034 | 0.34 | 3.8 | 293 |
| >Reverse \|Q6PGF7\|EXOC8_MOUSE Exocyst complex component 8 | 0.80 | 3 | 3 | 0.0042 | 0.42 | 2.7 | 716 |
| \|O70503\|DHB12_MOUSE Estradiol 17-beta-dehydrogenase 12 | 0.79 | 1 | 1 | 0.0032 | 0.32 | 5.1 | 312 |
| \|P18826\|KPB1_MOUSE Phosphorylase b kinase regulatory subunit alpha, skeletal muscle isoform | 0.78 | 2 | 2 | 0.0016 | 0.16 | 1.0 | 1241 |
| \|Q3V0C3\|FAM22_MOUSE Protein FAM22 | 0.75 | 2 | 1 | 0.0014 | 0.14 | 1.6 | 733 |
| >Reverse \|Q80WJ7\|LYRIC_MOUSE Protein LYRIC | 0.74 | 1 | 1 | 0.0017 | 0.17 | 1.9 | 579 |
| \|Q8CJ27\|ASPM_MOUSE Abnormal spindle-like microcephaly-associated protein homolog | 0.74 | 6 | 5 | 0.0016 | 0.16 | 0.6 | 3122 |
| \|Q9ERU9\|RBP2_MOUSE E3 SUMO-protein ligase RanBP2 | 0.72 | 2 | 1 | 0.0003 | 0.03 | 0.4 | 3053 |
| \|Q99LB7\|SARDH_MOUSE Sarcosine dehydrogenase, mitochondrial | 0.70 | 1 | 1 | 0.0011 | 0.11 | 0.9 | 919 |
| \|P48986\|NDF6_MOUSE Neurogenic differentiation factor 6 | 0.69 | 1 | 1 | 0.0030 | 0.30 | 12.5 | 337 |
| >Reverse \|Q91YE3\|EGLN1_MOUSE Egl nine homolog 1 | 0.68 | 2 | 1 | 0.0025 | 0.25 | 3.0 | 400 |
| \|Q80VA5\|S3TC2_MOUSE SH3 domain and tetratricopeptide repeats-containing protein 2 | 0.68 | 1 | 1 | 0.0008 | 0.08 | 0.5 | 1289 |
| \|E9Q557\|DESP_MOUSE Desmoplakin | 0.67 | 3 | 2 | 0.0007 | 0.07 | 0.7 | 2883 |
| >Reverse \|Q64435\|UD16_MOUSE UDP-glucuronosyltransferase 1-6 | 0.65 | 1 | 1 | 0.0019 | 0.19 | 2.8 | 531 |
| >Reverse \|Q9CZT8\|RAB3B_MOUSE Ras-related protein Rab-3B | 0.64 | 1 | 1 | 0.0046 | 0.46 | 5.0 | 219 |
| >Reverse \|P35276\|RAB3D_MOUSE Ras-related protein Rab-3D | 0.64 | 1 | 1 | 0.0046 | 0.46 | 5.0 | 219 |
| >Reverse \|P63011\|RAB3A_MOUSE Ras-related protein Rab-3A | 0.64 | 1 | 1 | 0.0045 | 0.45 | 5.0 | 220 |
| \|Q8BX70\|VP13C_MOUSE Vacuolar protein sorting-associated protein 13C | 0.63 | 1 | 1 | 0.0003 | 0.03 | 0.3 | 3748 |
| \|Q8BLA8\|TRPA1_MOUSE Transient receptor potential cation channel subfamily A member 1 | 0.63 | 1 | 1 | 0.0009 | 0.09 | 1.2 | 1125 |
| >Reverse \|P63038\|CH60_MOUSE 60 kDa heat shock protein, mitochondrial | 0.62 | 1 | 1 | 0.0017 | 0.17 | 1.6 | 573 |
| >Reverse \|O89019\|INVS_MOUSE Inversin | 0.60 | 1 | 1 | 0.0009 | 0.09 | 0.6 | 1062 |
| >Reverse \|O89019-2\|INVS_MOUSE Isoform 2 of Inversin |  |  |  |  |  |  |  |
| >Reverse \|O89019-3\|INVS_MOUSE Isoform 3 of Inversin |  |  |  |  |  |  |  |
| >Reverse \|O89019-4\|INVS_MOUSE Isoform 4 of Inversin |  |  |  |  |  |  |  |
| >Reverse \|O89019-5\|INVS_MOUSE Isoform 5 of Inversin |  |  |  |  |  |  |  |
| >Reverse \|O89019-6\|INVS_MOUSE Isoform 6 of Inversin |  |  |  |  |  |  |  |
| >Reverse \|O89019-7\|INVS_MOUSE Isoform 7 of Inversin |  |  |  |  |  |  |  |
